# Coded aperture snapshot spectral imaging fundus camera

**DOI:** 10.1038/s41598-023-39117-2

**Published:** 2023-07-25

**Authors:** Ruixuan Zhao, Chengshuai Yang, R. Theodore Smith, Liang Gao

**Affiliations:** 1 Department of Bioengineering, University of California Los Angeles, Los Angeles, CA 90095 USA; 2 Department of Ophthalmology, New York University School of Medicine, New York, NY USA

**Keywords:** Imaging and sensing, Biophotonics

## Abstract

Spectral imaging holds great promise for the non-invasive diagnosis of retinal diseases. However, to acquire a spectral datacube, conventional spectral cameras require extensive scanning, leading to a prolonged acquisition. Therefore, they are inapplicable to retinal imaging because of the rapid eye movement. To address this problem, we built a coded aperture snapshot spectral imaging fundus camera, which captures a large-sized spectral datacube in a single exposure. Moreover, to reconstruct a high-resolution image, we developed a robust deep unfolding algorithm using a state-of-the-art spectral transformer in the denoising network. We demonstrated the performance of the system through various experiments, including imaging standard targets, utilizing an eye phantom, and conducting in vivo imaging of the human retina.

## Introduction

Retinal imaging is crucial for the detection and management of ophthalmic diseases, such as age-related macular degeneration (AMD)^1^ and glaucoma^2^. The current standard-of-care retinal imaging technologies encompass color fundus photography, scanning laser ophthalmoscopes (SLO)^3^, and optical coherence tomography (OCT)^4^. Despite being extensively used in clinics, these techniques measure only spatial information of the retina. In contrast, spectral imaging captures light in three dimensions, i.e. acquiring both spatial coordinates (x, y) and wavelengths (λ) of a scene simultaneously. The rich information could be used to classify the underlying components of the object. Originally developed for remote sensing^5^, spectral imaging has gained increasing popularity in medical applications, including retinal imaging^6^. The overall rationale of using a spectral camera in retinal imaging is that the ocular tissue's endogenous optical properties, such as absorption and scattering, change during the progression of a retinal disease, and the spectrum of light emitted from tissue carries quantitative diagnostic information about tissue pathology.

To measure a spectral datacube (x, y, λ), conventional spectral imaging cameras rely on scanning, either in the spatial domain, such as using a slit scanning spectrometer^7^, or in the spectral domain, such as using a liquid-crystal-tunable-filter^8^. The scanning mechanism typically leads to a prolonged acquisition, making these techniques prone to motion artifacts. Furthermore, the data acquired from sequential measurements need to be registered in post-processing, a complicated procedure that is sensitive to motion and image signal-to-noise ratio (SNR). Particularly in retinal imaging, post-acquisition registration can result in artifacts due to tissue movement between successive images caused by arterial pulses as well as changes in the lens-eye geometry^9^. Additionally, it is challenging to keep a patient fixating on a target for an extended period of time.

A snapshot spectral imaging system can avoid all these problems and provide an ideal solution for obtaining retinal spectral data. In this category, representative techniques include computed tomographic imaging spectrometer (CTIS)^9,10^, the four-dimensional imaging spectrometer (4D-IS)^11^, image mapping spectrometer (IMS)^12,13^ and coded aperture snapshot spectral imagers (CASSI)^14,15^. Among these methods, only CASSI can measure a large-sized spectral datacube because it uses compressive sensing to acquire data^16,17^, leading to a high resolution along both spatial and spectral dimensions. CASSI uses a coded aperture (mask) and a dispersive element to modulate the input scene, and it captures a two-dimensional (2D), multiplexed projection of a spectral datacube. Provided that the spectral datacube is sparse in a given domain, it can faithfully be reconstructed with a regularization method. Essentially, just one spectrally dispersed projection of the datacube that is spatially modulated by the aperture code over all wavelengths is sufficient to reconstruct the entire spectral datacube^14^. Therefore, the acquisition efficiency of CASSI is significantly higher than non-compressive methods that directly map spectral datacube voxels to camera pixels, such as IMS.

In this work, we developed a spectral imaging device based on CASSI and integrated it with a commercial fundus camera. The resultant system can capture a 1180 × 1100 × 35 (x, y, λ) spectral datacube in a snapshot with a 17.5 μm and 15.6 μm resolution along the horizontal and vertical axes, respectively. The average spectral resolution is 5 nm from 445 nm to 602 nm. Moreover, to enable fast and high-quality image reconstruction, we developed a deep-learning-based algorithm. Once trained, our algorithm can reconstruct a megapixel CASSI image with only 60 s, a 20 times improvement compared with conventional iterative algorithms. We demonstrated the performance of the system through various experiments, including imaging standard targets, utilizing an eye phantom, and conducting in vivo imaging of the human retina. These experiments collectively showcase the system's capabilities and validate its effectiveness in capturing high-quality retinal images.

## System principle and method

### Optical setup and system model

**Figure 1:**
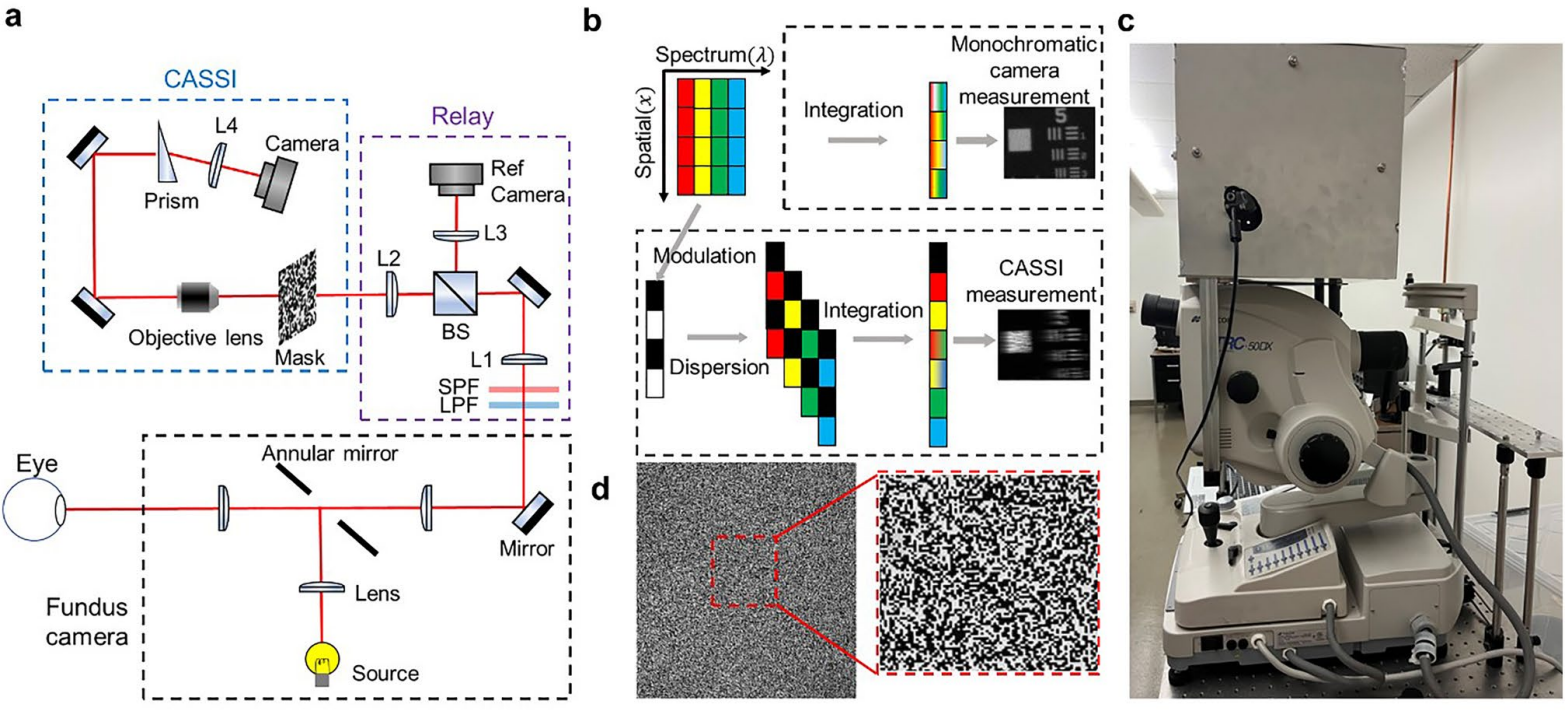
Coded aperture snapshot spectral imaging fundus camera. (**a**) Optical layout of the CASSI-fundus camera. (L1, L2: achromatic lens f = 50 mm; L3, L4: achromatic lens f = 100 mm; BS: 90/10 Beam splitter; LPF: long pass filter cutoff wavelength = 450 nm; SPF: short pass filter cutoff wavelength = 600 nm). (**b**) Image formation of monochromatic camera measurement and CASSI measurement. (**c**) The compact CASSI system mounted on the top image port of a commercial fundus camera (Topcon TRC-50DX). (**d**) Image of mask under 532 nm monochromatic light illumination (BW = 1 nm).

The schematic and photograph of the system are shown in Fig. 1a and c, respectively, where we couple a CASSI system to a commercial fundus camera (Topcon TRC-50dx) through its top image port. The illumination light is provided by the internal halogen lamp of the fundus camera. After being reflected by an annular mirror, a doughnut-shaped beam is formed and refocused onto the subject’s eye pupil, forming uniform illumination at the retina. The reflected light is collected by the same optics and forms an intermediate image at the top image port of the camera. This image is then passed to the CASSI system.

In the CASSI system, we use two achromatic lenses (AC254-50, f = 50 mm, Thorlabs) to relay the input image to a coded mask with a random binary pattern. The mask was fabricated on a quartz substrate with chrome coating by photolithography (Frontrange-photomask), as shown in Fig. 1d. The smallest coded feature of the pattern is sampled by approximately 2 × 2 pixels on the camera (11.6 μm), leading to a maximum solution of 1180 × 1100 pixels in the reconstructed image. The spectral range of the system is 450–600 nm, bandlimited by a combination of a 450 nm long pass filter (FELH0450, Thorlabs) and a 600 nm short pass filter (FESH0600, Thorlabs). Next, a 4f system consisting of an objective lens (4× /0.1 NA Olympus objective) and an achromatic lens (AC254-100, f = 100 mm, Thorlabs) relays the coded image to a CMOS image sensor (acA2500–60 um, Basler). To disperse the image, we position a round wedge prism (PS814-A, 10° Beam Deviation, Thorlabs) at the Fourier plane of the 4f relay system.

The CASSI measurement can be considered as encoding high-dimensional spectral data and mapping it onto a 2D space. As is shown in Fig. 1b, the coded aperture modulates the spatial information over the entire spectrum. The image right after the coded aperture is

1 $${f}_{1}\left(x,y;\lambda \right)={f}_{0}\left(x,y;\lambda \right)T\left(x,y\right),$$

where ${f}_{0}(x,y;\lambda )$ represents the spectral irradiance of the image at the coded aperture, and $T(x,y)$ denotes the transmission function of the coded aperture.

After passing the coded aperture, the spatially modulated information is spectrally dispersed by a prism. The spectral irradiance at the camera plane is

2 $${f}_{2}\left(x,y;\lambda \right)={f}_{0}\left(x+D\left(\lambda \right),y;\lambda \right)T\left(x+D\left(\lambda \right),y\right),$$

where $D\left(\lambda \right)$ denotes the nonlinear wavelength dispersion function of the prism.

The resultant intensity image captured at the detector plane is the superposition of multiple images of the spatially modulated scene at wavelength-dependent locations, which can be expressed as

3 $$Y\left(x,y\right)=\int {f}_{2}(x,y;\lambda )d\lambda .$$

Because the detector plane is spatially discretized with a pixel pitch of $\Delta$, $Y\left(x,y\right)$ is sampled across the entire 2D dimension of the detector plane. The measurement at the $(m,n)$ pixel can be written as

4 $$Y_{mn} = \mathop \smallint \limits_{m\Delta }^{{\left( {m + 1} \right)\Delta }} \mathop \smallint \limits_{n\Delta }^{{\left( {n + 1} \right)\Delta }} Y\left( {x,y} \right)dxdy\, = \,\iiint {{\text{f}}_{0} \left( {{\text{x}} + {\text{D}}\left( \lambda \right),{\text{y}};\lambda } \right){\text{T}}\left( {{\text{x}} + {\text{D}}\left( \lambda \right),{\text{y}}} \right){\text{rect}}\left( {\frac{{\text{x}}}{\Delta } - {\text{m}},\frac{{\text{y}}}{\Delta } - {\text{n}}} \right){\text{dxdyd}}\lambda } + {\text{g}}_{{{\text{mn}}}} ,$$

where ${g}_{mn}$ denotes the measurement noise at the $(m,n)$ pixel, and *rect* is a rectangular function.

After discretizing the spectral information into $L$ bands, the discrete measurements from the camera pixel can be written as

5 $${Y}_{mn}=\sum_{k=0}^{L-1}{f}_{(m-k)nk}{T}_{(m-k)n}+{g}_{mn}={\left(\phi f\right)}_{mn}+{g}_{mn},$$

where ${f}_{mnk}$ and ${T}_{mn}$ are the discretized representations of the source spectral irradiance and the coded aperture pattern, respectively.

### Deep unfolding reconstruction algorithm

Despite being simple in hardware, the image reconstruction of CASSI can be computationally extensive when using conventional iterative algorithms like Two-step iterative shrinkage/ thresholding (TwIST). Additionally, conventional iterative algorithms usually have two steps in each iteration: physical projection and hand-crafted priors^18–20^. The mismatch between the hand-crafted priors and real data often results in poor image quality. Recently, deep learning methods have been used in CASSI to improve reconstruction quality and reduce the time cost^21–23^. However, most deep learning methods fail to incorporate the physical information of the system, such as the mask pattern, into the model and treat reconstruction as a “black box” during the training phase. Therefore, these methods lack robustness and are prone to artifacts. To solve this problem, we combined the advantages of physical projection in conventional iterative reconstruction and the strong denoising ability of deep learning and developed a deep unfolding network for CASSI reconstruction. Because the mask information is mainly processed in the projection operation, our algorithm exhibits strong robustness to variations in mask patterns.

To reconstruct the original spectral datacube from the 2D CASSI measurement, we first vectorize the image and rewrite Eq. (5) in a matrix form:

6 $${\varvec{y}}=\boldsymbol{\Phi }{\varvec{f}}+{\varvec{g}}.$$

Given the measurement ***y*** and matrix $\boldsymbol{\Phi }$, there are two optimization frameworks to predict the original spectral scene ${\varvec{f}}$ : the penalty function method and the augmented Lagrangian (AL) method. Because the AL method outperforms the penalty function method, as shown in the previous studies^24–26^, we use the AL method to solve the above inverse problem:

7 $${\varvec{f}}=arg{min}_{{\varvec{f}}}\boldsymbol{\Psi }\left({\varvec{f}}\right)-{{\varvec{\lambda}}}_{1}^{T}\left({\varvec{y}}-\boldsymbol{\Phi }{\varvec{f}}\right)+\frac{{\gamma }_{1}}{2}{\Vert {\varvec{y}}-\boldsymbol{\Phi }{\varvec{f}}\Vert }_{2}^{2},$$

where $\boldsymbol{\Psi }\left({\varvec{f}}\right)$, ${{\varvec{\lambda}}}_{1}$, and ${\gamma }_{1}$ denote the prior regularization, Lagrangian multiplier, and penalty parameter, respectively. Equation (7) can be further written as

8 $${\varvec{f}}=arg{min}_{{\varvec{f}}}\boldsymbol{\Psi }\left({\varvec{f}}\right)+\frac{{\gamma }_{1}}{2}{\left\Vert {\varvec{y}}-\boldsymbol{\Phi }{\varvec{f}}-\frac{{{\varvec{\lambda}}}_{1}}{{\gamma }_{1}}\right\Vert }_{2}^{2}.$$

To solve Eq. (8), we adopt an alternating direction method of multipliers (ADMM) method^27–29^. According to ADMM, Eq. (8) can be stratified into two subproblems and solved iteratively

9 $${{\varvec{v}}}^{i}=arg{min}_{{\varvec{v}}}\boldsymbol{\Psi }\left({\varvec{v}}\right)+\frac{{\gamma }_{2}^{i}}{2}{\left\Vert {{\varvec{f}}}^{i-1}-{\varvec{v}}-\frac{{{\varvec{\lambda}}}_{2}^{i}}{{\gamma }_{2}^{i}}\right\Vert }_{2}^{2}$$

10 $${{\varvec{f}}}^{i}=arg{min}_{f}\frac{{\gamma }_{2}^{i}}{2}{\left\Vert {\varvec{f}}-{{\varvec{v}}}^{i}-\frac{{{\varvec{\lambda}}}_{2}^{i}}{{\gamma }_{2}^{i}}\right\Vert }_{2}^{2}+\frac{{\gamma }_{1}^{i}}{2}{\left\Vert {\varvec{y}}-\boldsymbol{\Phi }{\varvec{f}}-\frac{{{\varvec{\lambda}}}_{1}^{i}}{{\gamma }_{1}^{i}}\right\Vert }_{2}^{2},$$

where ${\varvec{v}}$ is an auxiliary variable, and the superscript *i* denotes the iteration index. Equation (9) is a classical denoising problem, which can be solved by a denoising prior such as total variation, wavelet transformation, or denoising network. Herein we use a deep unfolding network and a state-of-the-art spectral transformer for denoising^30^.

Equation (10) has a closed-form solution^29^, which is termed projection operation

11 $${{\varvec{f}}}^{i}={\left({\gamma }_{2}^{i}{\varvec{I}}+{\gamma }_{1}^{i}{\boldsymbol{\Phi }}^{T}\boldsymbol{\Phi }\right)}^{-1}\left[{{\varvec{\lambda}}}_{2}^{i}+{\gamma }_{2}^{i}{{\varvec{v}}}^{i}+{\boldsymbol{\Phi }}^{T}{\gamma }_{1}^{i}\left({\varvec{y}}-\frac{{{\varvec{\lambda}}}_{1}^{i}}{{\gamma }_{1}^{i}}\right)\right].$$

**Figure 2:**
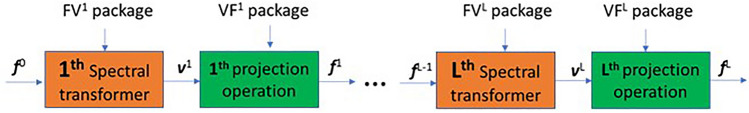
Deep unfolding algorithm for CASSI reconstruction. The projection operation is formulated in Eq. (11), and the spectral transformer is depicted in Ref.^30^. L is the total number of iterations.

Due to the special structure of $\boldsymbol{\Phi }$, which consists of a diagonal block matrix, as shown in Ref.^20^, Eq. (11) can be solved in one shot. Therefore, ${\varvec{f}}$ can be solved by multiple iterations of spectral-transformer (denoising) and projection operation, as shown in Fig. 2. Here ${{\varvec{f}}}^{0}$ is written as:

12 $${{\varvec{f}}}^{0}={\left({\gamma }_{2}^{i}{\varvec{I}}+{\gamma }_{1}^{i}{\boldsymbol{\Phi }}^{T}\boldsymbol{\Phi }\right)}^{-1}\left({\boldsymbol{\Phi }}^{T}{\varvec{y}}\right).$$

Figure 2 shows the iterative architecture of the deep unfolding algorithm. In the projection operation stage, ${{\varvec{f}}}^{i}$ is calculated from ${{\varvec{v}}}^{i}$ according to Eq. (11), where ${\varvec{y}}$, ${\gamma }_{1}^{i}$, ${\gamma }_{2}^{i}$, $\boldsymbol{\Phi }$, ${{\varvec{\lambda}}}_{1}^{i} ,\mathrm{and}$ ${{\varvec{\lambda}}}_{2}^{i}$ are inputs (For convenience, we use FV^i^ package to represent these inputs in Fig. 2). In the denoising operation stage, ${{\varvec{v}}}^{i}$ is calculated from ${{\varvec{f}}}^{i-1}$ according to Eq. (9), where ${\gamma }_{2}^{i}$, $\boldsymbol{\Phi }\boldsymbol{^{\prime}}$, and ${{\varvec{\lambda}}}_{2}^{i}$ are inputs (we use VF^i^ package to represent these inputs in Fig. 2). Additionally, the spectral transformer is used as the denoiser, which uses matrix $\boldsymbol{\Phi }\boldsymbol{^{\prime}}$ to guide the transformer. $\boldsymbol{\Phi }\boldsymbol{^{\prime}}$ is transformed by a convolution neural network (CNN) based on ${\gamma }_{2}^{i}$, $\boldsymbol{\Phi }$, ${\varvec{y}}$:

13 $$\boldsymbol{\Phi }\boldsymbol{^{\prime}}=CNN\left(concatenate\left({\gamma }_{2}^{i}{\varvec{I}},\boldsymbol{\Phi },\boldsymbol{ }{\varvec{t}}{\varvec{r}}{\varvec{a}}{\varvec{c}}{\varvec{e}}\left(\boldsymbol{\Phi }{\boldsymbol{\Phi }}^{T}\right)\boldsymbol{ }\right)\right).$$

The Lagrangian multiplier can be updated as

14 $${{\varvec{\lambda}}}_{1}^{i}={{\varvec{\lambda}}}_{1}^{i-1}-{\gamma }_{1}^{i}\left({\varvec{y}}-\boldsymbol{\Phi }{{\varvec{f}}}^{i-1}\right)$$

15 $${{\varvec{\lambda}}}_{2}^{i}={{\varvec{\lambda}}}_{2}^{i-1}-{\gamma }_{2}^{i}\left({{\varvec{f}}}^{i}-{{\varvec{v}}}^{i-1}\right),$$

and the penalty parameters ${\gamma }_{1}^{i}$, ${\gamma }_{2}^{i}$ can be trained in deep unfolding.

### Training

We used PyTorch^31^ to train our model on an NVIDIA RTX3090 GPU. The mean square error (MSE) was selected as the loss function. For training, we adopt an Adam optimizer^32^ and set the number of iterations in deep unfolding as four, the mini-batch size as one, and the spatial resolution as 256 × 256. Although we trained the model using images with 256 × 256 pixels, we can apply it to images of any dimensions after rescaling. The initial learning rate was set to 1×10^−4^. After the first five epochs, the learning rate decays at a rate of 0.9 every 15 epochs. The total number of epochs is 200, and the training time is about 60 hours.

## Results and discussion

### Rainbow object

**Figure 3:**
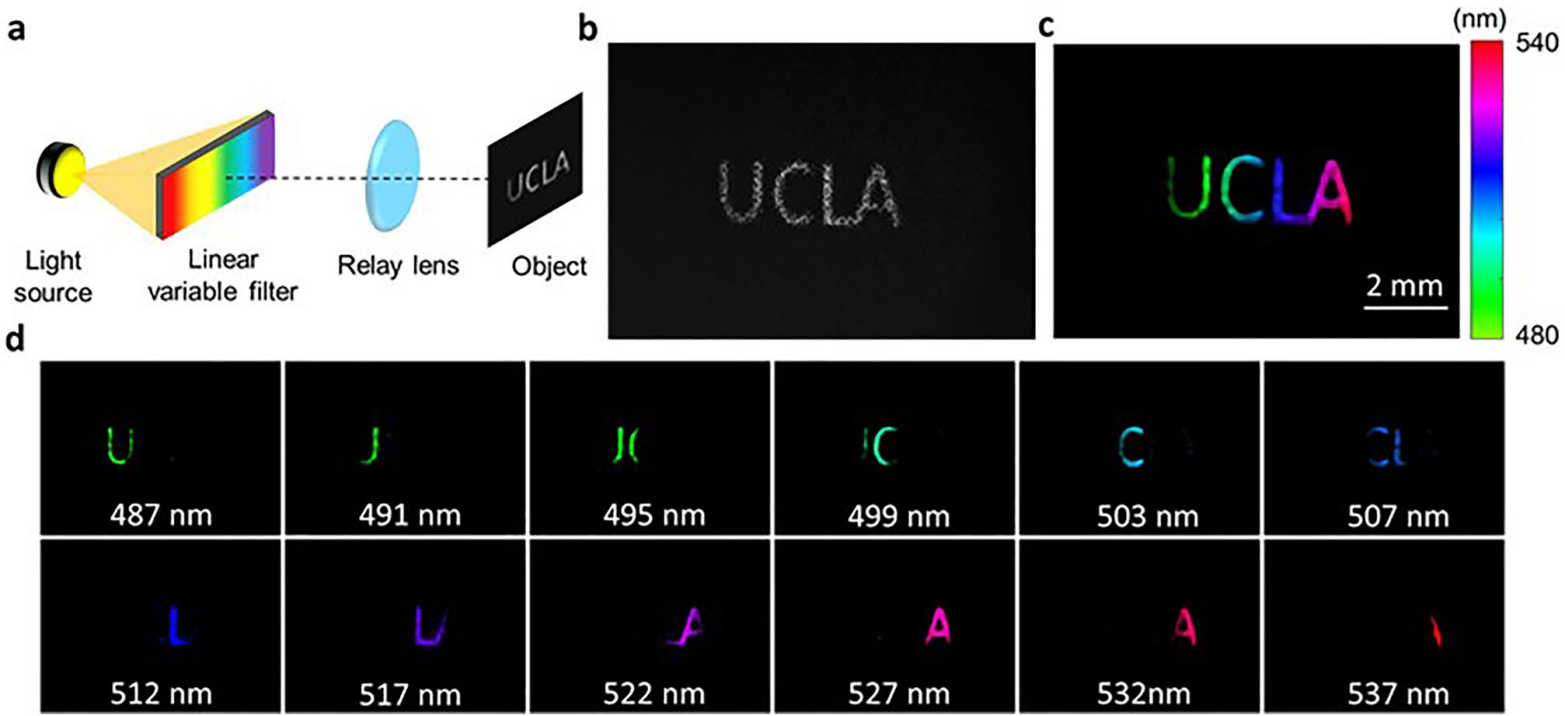
Spectral image of a letter object illuminated with rainbow light. (**a**) Illumination setup with a linear variable filter. (**b**) Raw measurement. (**c**) Pseudo-colored reconstructed panchromatic image. (**d**) Representative reconstructed spectral channel images.

We first validated our system by imaging an object illuminated with rainbow light. As shown in Fig. 3a, we positioned a linear variable visible bandpass filter (LVF) (88365, Edmund optics) in front of a broadband halogen light source. In LVF, the thickness of the coating varies linearly along one dimension of the filter, resulting in a linear and continuous variation in spectral transmission. The spectral resolution of the LVF is 7–20 nm. To project a broad spectrum to the field of view, we used a lens pair with the ratio of their focal lengths of 3.3:1 (MAP1030100-A, Thorlabs) to demagnify the linear filter and relayed it to an intermediate plane, where a letter object was located. Therefore, each lateral location of the object exhibited a distinct color. Figure 3b and c show the raw image and the reconstructed panchromatic image, respectively. Ten representative spectral images from a total of 35 channels are shown in Fig. 3d.

### Spatial and spectral resolutions

We quantified the spatial resolution of the system by imaging a USAF resolution target. We positioned the resolution target at the back focal plane of a lens that mimicked the crystalline lens in the eye and located the combined model in front of the fundus camera. Meanwhile, rather than using the theoretical mask pattern as a prior for reconstruction, we experimentally captured the coded mask image under uniform monochromatic illumination (Fig. 1d) and used this data to improve the reconstruction accuracy.

**Figure 4:**
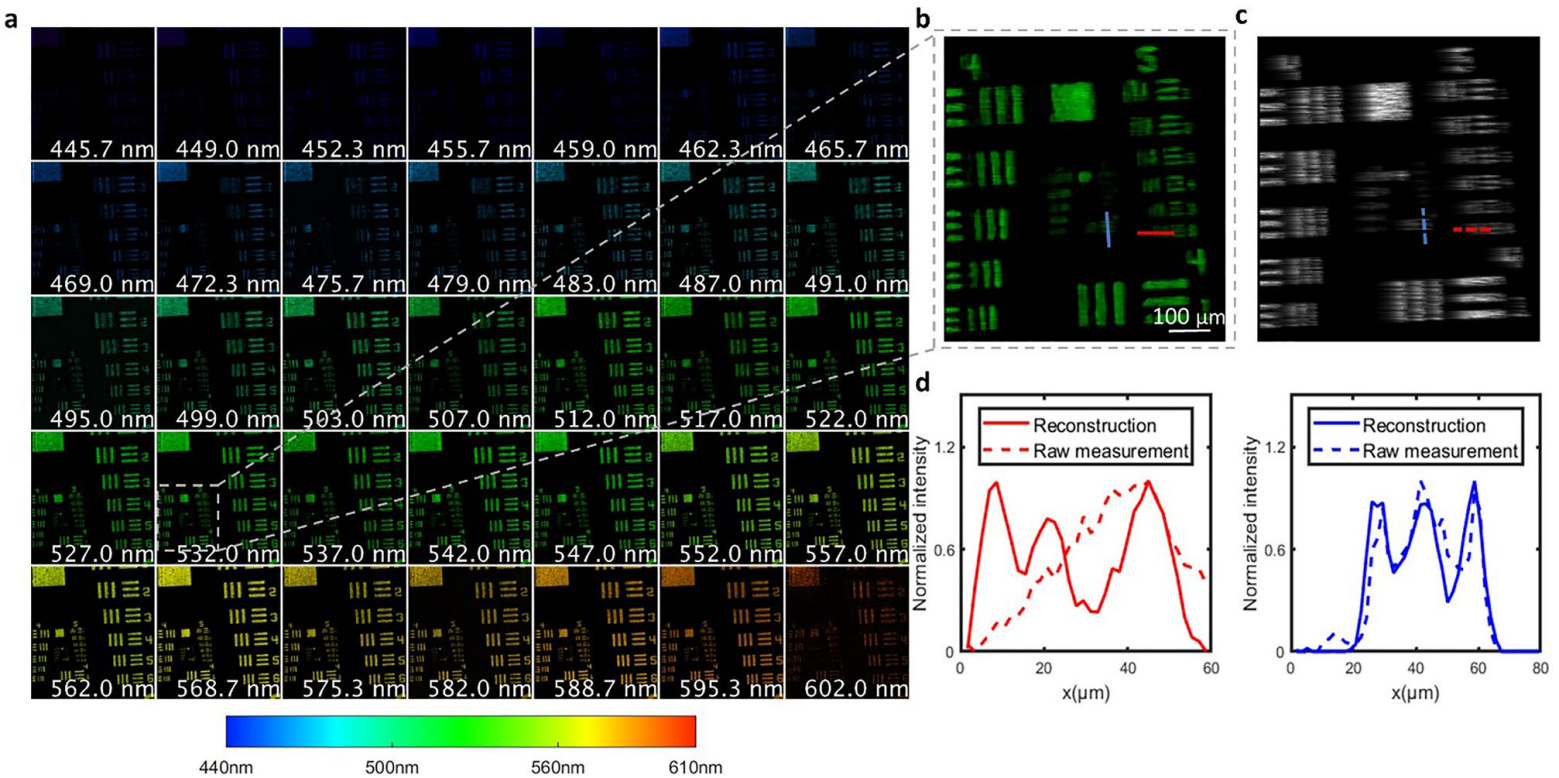
Quantification of spatial resolution. (**a**) Reconstructed spectral images of a USAF resolution target from 445 nm to 602 nm. (**b**) Zoomed-in view of the center part of the reconstructed image at 532 nm. (**c**) Zoomed-in view of raw measurement. (**d**) Intensities across the dashed lines in (**c**). The red dashed line corresponds to group 5 element 6 with 57 lp/mm. The blue dashed line corresponds to group 6 element 1 with 64 lp/mm.

We illuminated the USAF target in the transmission model with broadband light (450–600 nm). The reconstructed spectral images are shown in Fig. 4a, and a zoomed-in view of the 532 nm channel is shown in Fig. 4b. We also show the corresponding raw measurement in Fig. 4c, where the spatio-spectral crosstalk is clearly visible. After reconstruction, we successfully removed the spatial modulation pattern and restored high-resolution images in all spectral channels.

To quantify the spatial resolution, we zoomed in on the central part of the reconstructed 532 nm channel and plotted the intensities across the dashed lines in the image (Fig. 4d). The image contrast is defined as $\frac{{I}_{max}-{I}_{min}}{{I}_{max}+{I}_{min}}$, where $I$ is the intensity. We calculated the image contrast for each group of bars within the field of view (FOV). Given a threshold of 0.4, Group 6 element 1 horizontal bars and group 5 element 6 vertical bars are minimally resolvable features. The corresponding spatial resolution along the horizontal and vertical directions are 17.5 $\mathrm{\mu m}$ and 15.6 $\mathrm{\mu m}$, respectively.

The spectral resolution of the system is determined by the size of the smallest feature on the coded mask, the camera pixel size, and the spectral dispersion power of the prism^12^. In our system, the smallest coded feature corresponds to two camera pixels. The spectral dispersion across this distance determines the spectral resolution. Because the prism has nonlinear spectral dispersion, its spectral dispersion power varies as a function of wavelength. We measured the wavelength-dependent spectral resolution by imaging a blank FOV uniformly illuminated by monochromatic light of varied wavelengths. The dispersion distances (in pixels) between the coded mask images of adjacent wavelengths at four spectral bands are shown in Table 1. Because the smallest coded feature is mapped to two camera pixels, the spectral resolution is double the pixel dispersion.

**Table 1:** Wavelength-dependent spectral resolution.

Wavelength	Pixel dispersion at the camera plane	Spectral resolution
445–480 nm	1.67 nm/pixel	3.3 nm
480–510 nm	2.00 nm/pixel	4.0 nm
510–560 nm	2.50 nm/pixel	5.0 nm
560–602 nm	3.33 nm/pixel	6.6 nm

### Standard eye phantom

**Figure 5:**
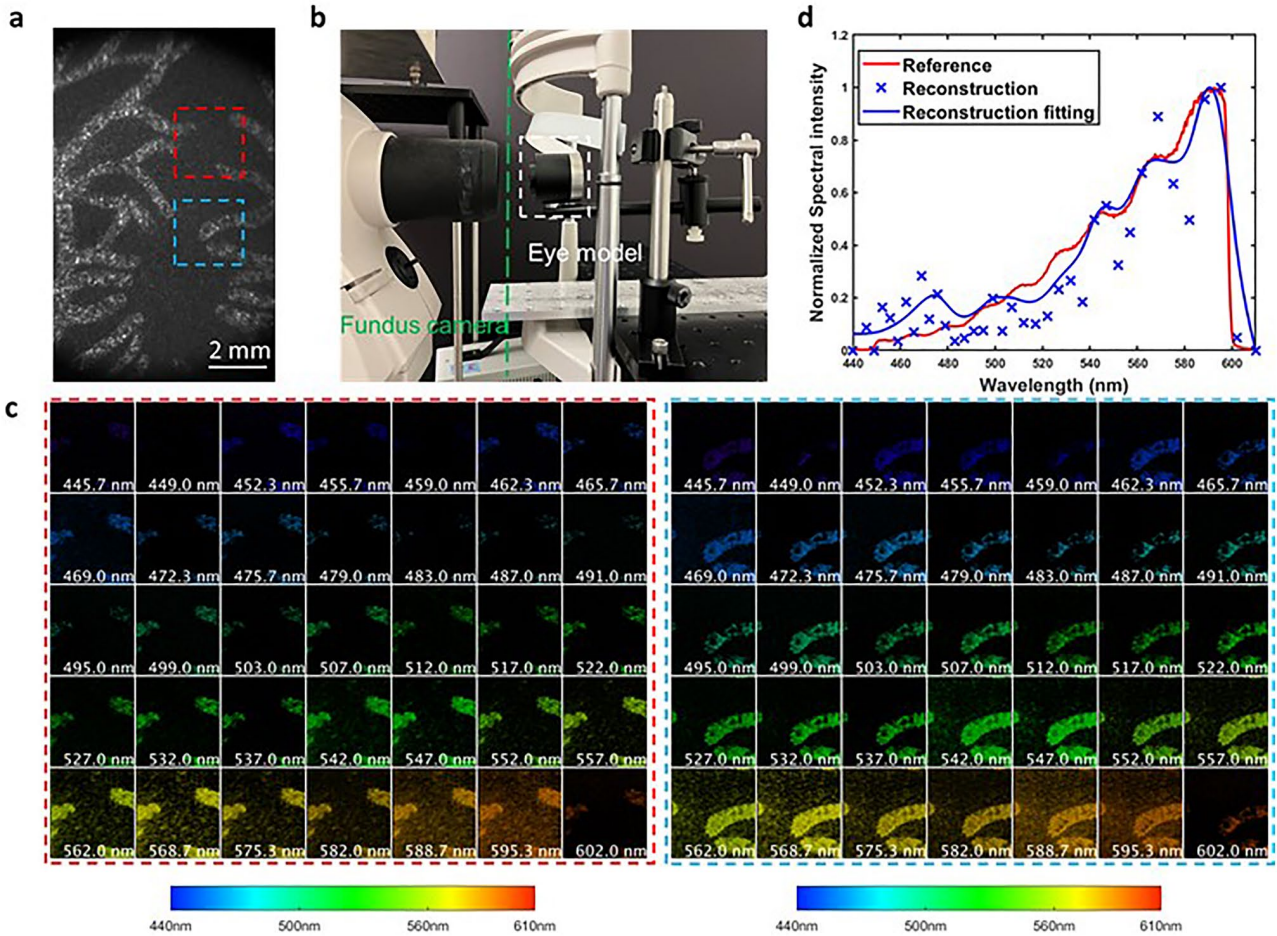
Spectral imaging of a standard eye model. (**a**) Reference image of the vascular structure in the eye model. The red and blue dashed boxes denote two regions of interest (ROIs). (**b**) Experimental setup. The eye model was placed in front of the objective lens of a fundus camera. (**c**) Reconstructed spectral channel images from 445 nm to -602 nm. (**d**) Reconstructed spectrum.

To further validate the system in retina imaging, we imaged a standard eye phantom (Wide field model eye, Rowe Technical design), which has both a realistic eye lens and a vasculature-like pattern painted at the back surface. A direct image of the retina obtained with a reference camera is shown in Fig. 5a. We positioned the eye phantom in front of the fundus camera (Fig. 5b), illuminated it with the camera’s internal halogen lamp, and captured the retina image using CASSI in a snapshot. The reconstructed spectral channel images of two ROIs are shown in Fig. 5c, showing a close resemblance to the corresponding regions in the reference image (Fig. 5a). Furthermore, to quantitatively evaluate the spectral accuracy, we measured the field-averaged spectrum using a benchmark fiber spectrometer (OSTS-VIS-L-25-400-SMA, Ocean Optics) as the reference. The CASSI reconstructed spectrum matches well with the ground truth (Fig. 5d).

### Spectral imaging of the retina in vivo

**Figure 6:**
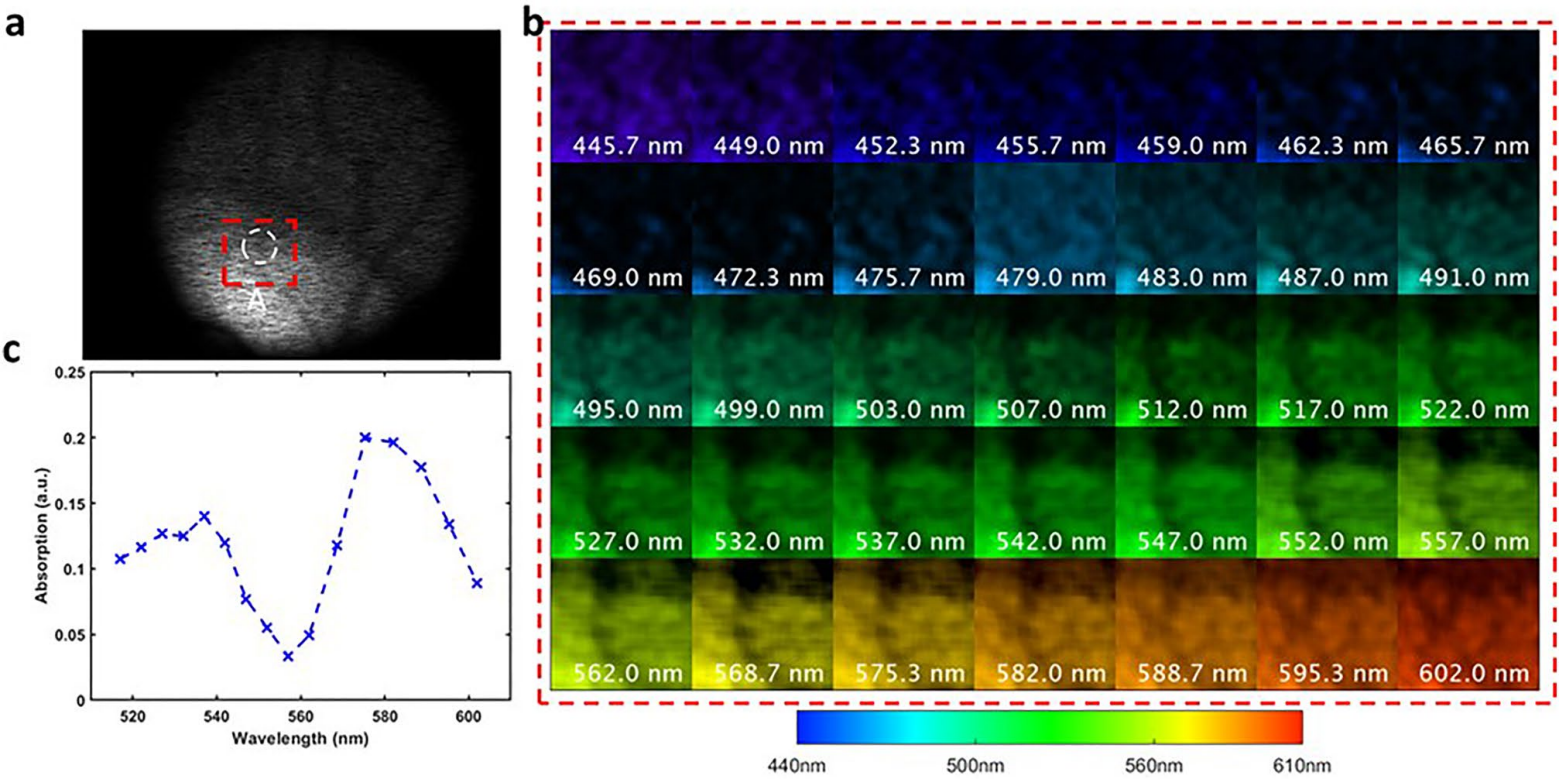
Spectral imaging of the retina around the optic disc in vivo. (**a**) Raw measurement of our fundus CASSI imaging system. The red box dashed box indicates the region of interest (ROI). (**b**) Reconstructed spectral image of the ROI. (**c**) Absorption spectrum of oxyhemoglobin in a retinal arteriole on the optic nerve.

To demonstrate our system in vivo, we imaged the retina of a 23-year-old healthy female volunteer. Before the experiment, the subject's pupil was dilated using mydriatic (Mydriacyl, 1%). We operated the camera in the reflectance mode and utilized the internal light source of the camera for illumination. The snapshot measurement and reconstructed spectral image are displayed in Fig. 6a and b, respectively.

Next, we calculated the reflectance spectrum ${{\varvec{S}}}_{{\varvec{r}}}$ at a vessel location by averaging the pixels’ spectra in the circled area (A in Fig. 6a). To compute the absorption spectrum, we measured the illumination spectrum ${{\varvec{S}}}_{{\varvec{i}}}$ of the fundus camera's internal lamp by placing a white paper in front of the retinal camera's front lens and averaging the reflectance spectra of pixels within the field of view. The absorption spectrum ${{\varvec{S}}}_{{\varvec{a}}}$(Fig. 6c) was then calculated by subtracting the normalized reflectance spectrum ${{\varvec{S}}}_{{\varvec{r}}}$ from the normalized lamp’s illumination spectrum ${{\varvec{S}}}_{{\varvec{i}}}:$

16 $${{\varvec{S}}}_{{\varvec{a}}}=\frac{{{\varvec{S}}}_{{\varvec{i}}}}{\mathbf{m}\mathbf{a}\mathbf{x}({{\varvec{S}}}_{{\varvec{i}}})}-\frac{{{\varvec{S}}}_{{\varvec{r}}}}{\mathbf{max}\left({{\varvec{S}}}_{{\varvec{r}}}\right)}.$$

The resulting spectrum (Fig. 6c) exhibits two prominent peaks, which correspond to the peak absorptions of oxyhemoglobin at approximately 540 nm and 575 nm^33^.

## Discussion

Figure 3 demonstrates the capability of our system in resolving the overlap between spatial and spectral information. By encoding each lateral coordinate of the planar letter object with a unique color, we successfully reconstructed the images of individual letters in different spectral channels (487 nm, 499 nm, 517 nm, and 527 nm for the letter 'UCLA'). Our deep unfolding reconstruction network effectively removes the spatial modulation pattern caused by the coded aperture in all spectral channels.

To further enhance the spatial resolution of our proposed system, several strategies can be employed. One approach is to utilize an objective lens with a higher numerical aperture (NA). Additionally, using a mask with smaller features and a camera with a smaller pixel size while maintaining the 2 × 2 sampling rate can also improve spatial resolution. Another contributing factor to the quality of reconstruction is the camera sensor. In our system, we employed a machine vision camera with limitations such as low quantum efficiency and high readout noise. Overcoming these limitations can be achieved by selecting a scientific monochromatic sensor with high quantum efficiency and superior noise performance. Such improvements are particularly beneficial for clinical applications.

Figures 5 and 6 present the reconstruction results of the eye phantom and in vivo retina, respectively. The spectrum of the eye phantom aligns well with the ground truth illumination light source. Moreover, we successfully acquired the absorption spectral signatures of oxyhemoglobin through in vivo imaging. In terms of image quality, the reconstructed vascular structure in the eye phantom exhibits higher contrast compared to in vivo imaging. This is due to the relatively high reflection of the painting material in the eye phantom, resulting in a distinct contrast between the vascular structure (bright regions) and the background (dark regions). However, in the case of in vivo imaging, the low reflectance of the human retina poses a more challenging problem for conventional iterative algorithms like TwIST^18^. Leveraging the denoising capabilities of the spectral transformer module, our deep unfolding network demonstrates robustness under various imaging conditions.

Furthermore, our deep-learning-based algorithm can reconstruct the entire megapixel datacube within 60 s, a significant improvement compared to the conventional iterative algorithms that would typically require 20 min. While compromising the snapshot capability and capturing multiple shots with different mask patterns is another method to improve performance, it necessitates additional computational time and hardware components such as a spatial light modulator (SLM) for dynamically changing the mask patterns^34^.

## Conclusions

In summary, we developed a snapshot spectral retinal imaging system by integrating a CASSI system with a fundus camera. We also developed a deep unfolding method for fast and high-quality image reconstruction. The resultant system can acquire a large-sized spectral datacube in the visible light range in a single exposure. The system performance has been demonstrated with standard targets, eye phantom and human retina imaging test. In the in vivo human retinal imaging experiment, the absorption spectral signatures of oxyhemoglobin were successfully acquired by using our proposed system. Seeing its high-resolution snapshot imaging advantage, we expect our method can find broad applications in retinal imaging.

## Data Availability

Data underlying the results presented in this paper may be obtained from the corresponding author upon reasonable request.
